# Beyond Primum Non Nocere: Therapeutic Injury, Comparative Harm Management, and the Ethics of Modern Medicine

**DOI:** 10.1111/jep.70643

**Published:** 2026-10-04

**Authors:** Boris Nikolic

**Affiliations:** ^1^ Department of Philosophy Heinrich‐Heine‐Universität Düsseldorf Düsseldorf Germany

**Keywords:** clinical decision‐making, comparative harm management, harm, medical ethics, non‐maleficence, primum non nocere, proportionality

## Abstract

**Rationale, Aims and Objective:**

The maxim primum non nocere (“first, do no harm”) remains emblematic of medical ethics, yet much of modern therapeutics accepts foreseeable and sometimes near‐certain injury in pursuit of benefit: surgery injures tissue, chemotherapy produces systemic toxicity, and ablation and embolization achieve their effect through controlled destruction. This paper asks how such therapeutic injury is to be ethically understood, and whether non‐maleficence still describes the operative morality of clinical practice.

**Method:**

The paper proceeds by critical conceptual and ethical analysis rather than empirical study, situating the question within the literature that has sought to refine non‐maleficence—principlism, the doctrine of double effect, proportionality reasoning, threshold deontology, and the ethics of risk.

**Results:**

Contemporary medicine is argued to operate not through literal non‐maleficence but through comparative harm management: the selection among competing, foreseeable harms under uncertainty. A distinct and growing class of interventions produces injury that is not a side effect but the therapeutic mechanism itself, a case the inherited frameworks address only obliquely. Comparative harm management is shown to describe the architecture of clinical decision‐making rather than to add a further criterion of justification such as proportionality.

**Conclusions:**

Non‐maleficence retains a regulative and cautionary value but no longer describes the operational morality of modern medicine. The physician is best understood not as a guarantor of freedom from harm but as a steward of proportionate therapeutic injury under conditions of uncertainty, vulnerability, and competing obligations.

## 1 Introduction

Few ethical principles in medicine are as universally recognised as the maxim ‘primum non nocere.’ The phrase functions both as a moral aspiration and as a public symbol of physician restraint. Yet contemporary medical practice reveals a profound paradox: many accepted therapies intentionally or foreseeably produce harm in order to prevent greater harms associated with disease progression.

Modern medicine routinely accepts foreseeable and sometimes near‐certain injury as an intrinsic component of treatment itself. Surgery intentionally damages tissue, chemotherapy knowingly produces systemic toxicity, embolization deliberately induces ischemia, and tumour ablation achieves therapeutic aims through controlled destruction.

The central ethical problem is therefore not whether medicine can avoid harm, but how foreseeable therapeutic harm can be ethically justified. Contemporary clinical practice functions through comparative harm management: physicians accept proportionate and contextually justified injuries in order to prevent greater harms associated with disease progression, disability, or death.

This paper argues that non‐maleficence remains morally important but no longer functions as an absolute operational principle. Instead, contemporary medicine relies upon practical judgement capable of balancing competing harms, uncertain outcomes, patient autonomy, and proportional therapeutic goals.

One clarification belongs at the outset, because it shapes everything that follows. Comparative harm management is not a calculus the physician performs upon a patient; it is a judgement reached with one. The harms weighed are borne by a person who may accept them, decline them, or accept some and refuse others, and whose own ranking of the goods at stake is not an input the physician can supply. Consent is therefore not a procedural formality appended to the analysis but one of its conditions: an injury that would otherwise be a wrong becomes a treatment in part because it is authorised by the person who undergoes it. The Kantian form of this point is developed below, but it should be visible from the start.

### 1.1 Therapeutic Injury in Contemporary Medicine

Modern medicine contains countless examples in which therapeutic intervention necessarily produces injury in order to prevent greater harm caused by disease. In interventional radiology, pneumothorax is knowingly accepted during transthoracic lung biopsy or thermal ablation procedures. Embolization intentionally creates ischemia to control hemorrhage or destroy tumours. Cryoablation deliberately induces tissue necrosis [1].

Similar examples exist throughout medicine. Surgical procedures universally involve intentional tissue injury. Chemotherapy knowingly damages healthy rapidly dividing cells in pursuit of malignancy control; this trade‐off between therapeutic benefit and systemic toxicity is well established in the oncological literature, and the quality‐of‐life consequences of cytotoxic treatment remain a central ethical concern even in advanced disease [2, 3]. Radiation therapy produces collateral tissue exposure. Creation of arteriovenous fistulas for hemodialysis may predictably produce steal syndrome or distal ischemic complications, including ischemic monomelic neuropathy [4, 5]. Neurosurgical treatment of intracranial lesions frequently requires what has been termed ‘maximal safe resection’: an explicit acceptance that achieving adequate tumor control may necessitate operating into eloquent cortical regions, accepting the risk of permanent neurological deficit as a proportionate therapeutic trade‐off [6, 7].

These examples differ philosophically from accidental complications. A wire perforation during angiography may represent an unintended adverse event. By contrast, tissue destruction during ablation is itself the therapeutic mechanism. Modern medicine therefore frequently functions through controlled injury.

Importantly, these harms are not tolerated because they occasionally accompany treatment. In many interventions they are structurally constitutive of therapy itself. This distinction between accidental complication and therapeutically intended injury forms the conceptual center of the present argument.

None of this suggests that injurious treatment is a modern invention. Trepanation, cautery, blistering and above all therapeutic bloodletting were practised for centuries and were plainly injurious; each was justified, within the physiological theory of its time, as restoring a disturbed balance or expelling a morbid agent. The difference is not that earlier medicine avoided harm while contemporary medicine inflicts it, but that the relation between the injury and the benefit has changed its epistemic status. Bloodletting was believed to help through a mechanism that did not exist. In thermal ablation or therapeutic embolization the injury is the mechanism, it is known to be so, and its extent is titrated deliberately against the disease it is meant to destroy. What is new is not harm in medicine but harm as a validated instrument, delivered with precision and defended on evidence [8].

## 2 The Ethical Baseline Problem

Medical interventions are rarely compared against perfect health. Rather, they are compared against the anticipated natural course of disease. A patient with untreated malignancy may face progressive suffering, organ failure, or death. A patient without dialysis access may die from renal failure. A patient with untreated intracranial hemorrhage may suffer catastrophic neurological decline.

In such contexts, the ethical question is not whether harm occurs, but which configuration of harms is ethically preferable. Medicine therefore operates through comparative harm management rather than pure harm avoidance. Therapeutic decisions frequently involve accepting lesser or more proportionate harms to prevent greater harms associated with disease progression.

This insight significantly weakens literal interpretations of ‘do no harm.’ If interpreted rigidly, the principle would prohibit much of modern medicine and potentially condemn patients to the harms imposed by untreated disease itself.

### 2.1 Refining ‘Do No Harm’: The Existing Literature

The claim that medicine cannot operate through literal harm‐avoidance is not itself new, and any contribution in this area must be situated against a substantial literature that has sought to refine, rather than abandon, the principle of non‐maleficence. At least three strands of that literature bear directly on the present argument.

First, the standard analytic treatment of non‐maleficence already rejects the literal reading. Beauchamp and Childress [9] separate non‐maleficence from beneficence and hold that the obligation not to inflict harm, though generally more stringent than the obligation to confer benefit, is prima facie rather than absolute. On their account the operative standard is not the avoidance of harm but due care: harm may be imposed where a proportionate good is sought, competently pursued, and justified by the anticipated benefit relative to the risk. This is, in substance, a detriment–benefit logic close to what the present paper calls comparative harm management, and it is anticipated by Frankena's [10] ordering of the duties of beneficence, in which not inflicting harm ranks ahead of preventing or removing it. An argument that proposes proportional harm‐balancing without engaging these accounts risks presenting a settled position as a discovery.

A further distinction within this literature bears directly on the standing of the maxim: Gert, Culver and Clouser [11]) separate moral rules, which are prohibitions and are morally required, from moral ideals, which are aspirations and are morally encouraged; on that account injunctions such as ‘do not cause pain’ and ‘do not disable’ belong to the first class rather than the second. Read as a prohibition, primum non nocere is stricter than the aspirational reading commonly attributed to it, and the difficulty addressed here becomes sharper rather than softer: a prohibition that contemporary practice violates as a matter of routine is not a demanding ideal imperfectly met but a rule whose scope has been misdescribed. The argument developed below is compatible with either reading, but it is the prohibitive reading that makes redescription unavoidable.

Second, the doctrine of double effect (DDE) is the most developed attempt to specify when foreseeable harm is permissible. Its classical conditions—that the act not be intrinsically wrong, that the bad effect not be the means to the good, that the harm be foreseen but not intended, and that there be proportionate reason—supply precisely the proportionality requirement invoked in this paper [12, 13]. The tradition is also contested: McIntyre [14] argues that the intention–foresight distinction cannot carry the moral weight assigned to it, and that the proportionality condition does the real justificatory work. Both the doctrine and its critics are therefore part of the refinement literature that any account of therapeutic harm must address.

Third, a specifically medical literature has examined ‘primum non nocere’ as such. Historical scholarship has shown the maxim to be neither Hippocratic in origin nor semantically straightforward [15, 16], while Jonsen [17] and, in this journal, Sharpe [18] have argued that ‘do no harm’ has always functioned as a regulative ideal requiring interpretation rather than as a literal prohibition, while principlism [19], placing non‐maleficence among the four prima facie principles, likewise treats it as one consideration to be weighed against beneficence, respect for autonomy, and justice. The field, in short, set aside literal non‐maleficence some time ago.

Locating the argument in this way sharpens what remains to be said. If proportionate, weighed harm is already the consensus, the contribution here cannot be the bare claim that medicine accepts justified harm. It lies instead in a structural feature the inherited refinements do not squarely address: a growing class of interventions—thermal and chemical ablation, therapeutic embolization, deliberate infarction, maximal safe resection—in which the injury is not a side effect of treatment but its very mechanism. For such cases the doctrine of double effect's pivotal distinction, between harm as a means and harm as a merely foreseen consequence, is not just hard to apply but structurally inapt, since the injury simply is the means. It is here that the due‐care standard and the classical proportionality conditions underdetermine the ethics, and where an explicit account of comparative harm management—weighing constitutive therapeutic injury against the harms of untreated disease under conditions of epistemic uncertainty—does work the existing literature leaves undone.

## 3 Medicine as a Moral Enterprise: Beneficence and the Patient's Good

If the analytic tradition treats non‐maleficence as one prima facie principle among several, a second tradition questions whether it is a freestanding principle at all. Pellegrino and Thomasma [20] conceive medicine as a moral enterprise constituted by its end—the good of the patient—rather than by a set of external side‐constraints. On this view the physician's primary obligation is beneficence, the active pursuit of the patient's good across its several dimensions: the medical good, the patient's own perception of their good, the good of the patient as a person, and the good as such. Non‐maleficence is not an independent commandment but the inner limit of beneficence: one does not harm because, and insofar as, harming fails to serve the patient's good.

This framing bears on the present argument in two ways. First, it explains why ‘do no harm’ was never intended as an absolute prohibition: within a beneficence‐centred ethic the relevant question is always whether an intervention, harms included, serves the patient's good on balance. Second, it relocates the moral work—from avoiding harm to determining what the patient's good requires under the circumstances. To that extent Pellegrino and Thomasma already concede the structure defended here, in which therapeutic injury is justified when it is instrumental to, or constitutive of, the patient's good. What their framework leaves open is the harder question this paper presses: how competing harms are to be weighed when the injury is itself the means by which the good is pursued.

### 3.1 Therapeutic Injury and the Limits of Double Effect

The doctrine of double effect deserves closer scrutiny than its frequent invocation suggests, because the interventions at issue here strain it at its most load‐bearing point. The doctrine permits an act with a foreseen bad effect when four conditions hold: the act is not intrinsically wrong; the agent intends only the good effect; the bad effect is not the means by which the good is achieved; and the good is proportionate to the bad [12, 13]. The third condition—that the harm not be the means—does the decisive work.

Many therapeutic injuries fail this condition not marginally but in principle. When an interventional radiologist embolizes a tumour's blood supply, the ischemia is not a regrettable accompaniment of the cure; the ischemia is the cure. When a surgeon undertakes maximal safe resection into eloquent cortex, the tissue removal is the therapeutic act. When thermal ablation induces coagulative necrosis, the necrosis is the mechanism. In each case the harm is the means to the benefit, which is just what the doctrine forbids. A defender may reply that the true object of intention is the disease, not the healthy tissue, and that the injury is ‘directed’ at pathology; but this redescription either dissolves the means–end distinction the doctrine requires or proves too much, licensing any harm that can be redescribed as aimed at a further good [14].

The lesson is not that double effect is worthless but that it is the wrong instrument for constitutive therapeutic injury. Its natural home is the foreseen side effect—the respiratory depression that may accompany adequate palliative sedation, the collateral irradiation of healthy tissue—where intention and mechanism genuinely come apart. Where they do not, the entire ethical weight falls on the doctrine's fourth condition, proportionality, and the doctrine itself offers no account of how proportionality is to be judged. Closing that gap is the task of the sections that follow.

### 3.2 Proportionality Beyond Double Effect

Proportionality is not peculiar to the doctrine of double effect; it is a recurring structure across applied ethics, and its medical uses are more developed than a single invocation suggests. Three are salient. In end‐of‐life care, the long tradition distinguishing proportionate from disproportionate means—historically framed as ‘ordinary’ versus ‘extraordinary’ treatment—deems an intervention obligatory or optional according to the ratio of its expected benefits to its burdens for this patient, an explicitly comparative, burden‐weighing judgement rather than a rule of harm avoidance [9].

In critical care and surgery, proportionality governs decisions to initiate, escalate, or withdraw aggressive interventions whose burdens—organ support, repeated operation, prolonged suffering—are weighed against the probability and magnitude of benefit. In public‐health ethics, proportionality is a named justificatory condition: coercive or harmful measures such as quarantine or compulsory treatment are permissible only when the anticipated benefit is weighty, the measure necessary, and the imposed burden proportionate to the threat addressed [21]. Across all three domains the operative logic is not ‘do no harm’ but ‘impose no more harm than the avoidable greater harm warrants.’

These literatures matter here because they show that comparative harm management is not a coinage but the implicit grammar of several mature areas of medical ethics. The contribution of this paper is to make that grammar explicit and to extend it to a case the existing domains treat only obliquely: interventions in which the imposed burden is not a side effect of treatment but its therapeutic mechanism.

### 3.3 Threshold Deontology and the Imposition of Harm

A deeper challenge to any comparative account comes from deontology. If there are genuine constraints against harming, then trading harms off against benefits is precisely what morality forbids, and a physician may not injure one interest simply because doing so averts a greater injury elsewhere. The contemporary debate over the structure and limits of such constraints is therefore directly relevant. Thomson's [22] analysis of permissible and impermissible harmings, and Kamm's [23] intricate account of when harm may be done as a means, as a side effect, or to a person who is also thereby aided, supply the tools for distinguishing therapeutic injury from the harms deontology prohibits.

Two features of the medical case ease the deontological worry. First, the person harmed is, in the standard case, the very person benefited; therapeutic injury is not the sacrifice of one party for another, which is where constraints bite hardest. Second, even theorists who defend robust constraints rarely hold them absolute. Kagan [24] presses the question how constraints could be anything but threshold‐bounded, and so‐called threshold deontology [25] holds that a constraint may be overridden once the harm prevented becomes sufficiently grave. If that is right, the distance between deontologist and consequentialist over therapeutic harm narrows sharply: both can accept that proportionate injury inflicted on a patient, with that patient's consent, to prevent a far greater harm to the same patient, lies on the permissible side of the line.

### 3.4 Risk, Uncertainty, and the Ethics of Trade‐Offs

Comparative harm management is exercised under uncertainty, and this is not an incidental complication but a defining one. Here the ethics of risk supplies resources that a purely clinical vocabulary lacks. Hansson [26] argues that the standard moral theories, framed for choices with known outcomes, are poorly suited to decisions whose outcomes are probabilistic, and that the permissibility of imposing risk turns on factors—consent, the distribution of risks and benefits, the availability of less hazardous alternatives—rather than on expected value alone. Rescher [27] likewise treats risk evaluation as an irreducibly normative undertaking rather than a technical computation.

Applied to therapeutic injury, this yields two corrections to a naive proportionality. First, what is weighed is seldom a harm against a benefit but a distribution of possible harms against a distribution of possible benefits, so that reversibility, the steepness of the worst case, and the patient's own attitude to risk enter the judgement essentially. Second, the relevant probabilities are ordinarily known with less precision than the decision requires. Clinicians are not without expectations: for most established interventions the likeliest outcome is well characterised at the level of populations, and it is on such evidence that the offer of treatment rests. What is frequently unavailable is a dependable estimate for this patient, whose comorbidity, anatomy or prior treatment may place him outside the population from which the estimate was drawn. The physician's obligation is therefore procedural as much as substantive: to deliberate well, to disclose what is and is not known, and to share the decision with the patient whose risk it finally is. Comparative harm management is, in this light, a discipline of judgement under uncertainty rather than an arithmetic of outcomes.

### 3.5 Proportionality and Comparative Harm

Throughout this paper, proportionality refers to the ethical justification of foreseeable therapeutic harm when the anticipated therapeutic good is sufficiently weighty, probable, and unattainable through substantially less injurious alternatives.

Proportionality therefore requires consideration of several factors:

- magnitude of anticipated benefit,
- severity and reversibility of harm,
- probability of therapeutic success,
- availability of less harmful alternatives,
- patient goals and values,
- temporal urgency, and
- the natural trajectory of untreated disease.

Proportionality is not reducible to mathematical calculation. Rather, it represents a context‐sensitive ethical judgement regarding whether a foreseeable injury remains morally justified relative to the harms likely imposed by disease itself.

### 3.6 Why Comparative Harm Management Is Not Merely Proportionality

A skeptical reader may suspect that comparative harm management is proportionality under a new name. The two are not the same, and the difference is worth stating precisely. Proportionality is a criterion of justification: a test applied to a particular intervention to determine whether a foreseeable harm is licensed by a sufficiently weighty, probable, and otherwise unobtainable good. It presupposes a default—that harm is not to be done—and specifies the conditions under which that default may be overridden in the individual case. Comparative harm management, by contrast, is not a test but a description of the architecture of clinical decision‐making. Its claim concerns what the basic unit of medical choice actually is: not the avoidance of harm with occasional justified exceptions, but the selection among competing configurations of harm under uncertainty.

The distinction becomes concrete once one notices that medicine has no harm‐free option to which it might default. The alternative to a harmful intervention is rarely the absence of harm; it is the harm of untreated disease, of a foregone diagnosis, of a deferred decision. Every branch of the clinical decision tree carries an expected burden, including the branch labelled ‘do nothing.' Proportionality, framed as the justified exception to a harm‐avoiding default, quietly assumes a baseline that clinical reality does not supply. Comparative harm management drops the assumption: there is no privileged harmless option against which the others stand as exceptions, and so the physician is always choosing among harms, never between harm and its absence. Proportionality then reappears, but in a subordinate role—as one of the criteria by which a particular chosen harm is justified once the field of options has been laid out.

Constitutive therapeutic injury sharpens the point. The justificatory frameworks—double effect above all—are built for harm that is separable from benefit: a cost incurred alongside, or as a foreseen consequence of, a beneficial act, which the benefit must then outweigh. But in embolization, ablation, deliberate infarction, and resection, the injury is not separable from the benefit; it is the mechanism through which the benefit is produced. There is no independent harm to be set against an independent good, because the harm and the therapeutic effect are one event under two descriptions. For such interventions proportionality cannot even be stated in its usual form, since it presupposes two distinct quantities to be weighed. What can be stated is a comparison between this configuration of mechanism‐and‐effect and the configurations available elsewhere, the configuration of untreated disease included.

The thesis is therefore structural rather than terminological. Proportionality is something a clinician does to a candidate action; comparative harm management is the shape of the situation within which candidate actions arise. As modern therapeutics comes to rely ever more heavily on injury‐as‐mechanism—and as algorithmic decision support increasingly formalises the comparison of outcome distributions—this structural feature grows harder to assimilate to the older picture of a harm‐avoiding practice that tolerates justified exceptions. Comparative harm management is offered not as a rival criterion to proportionality but as a more accurate description of the terrain on which proportionality, prima facie duties, and threshold constraints all operate.

### 3.7 Utilitarian and Consequentialist Frameworks

Utilitarian philosophy provides one intuitive explanation for therapeutic injury. Under consequentialist reasoning, foreseeable harms may be justified when they reduce overall suffering or maximise aggregate benefit. Chemotherapy toxicity, procedural morbidity, or surgical injury become ethically acceptable because they prevent greater harms associated with disease progression.

Yet medicine cannot rest entirely upon utilitarian foundations. Pure utilitarian reasoning risks treating patients as interchangeable units within aggregate calculations of social utility. Such reasoning may justify disproportionate sacrifice, excessive interventionism, or therapeutic aggression insufficiently attentive to dignity, identity, or autonomy.

Moreover, medicine operates under conditions of profound epistemic uncertainty. Physicians rarely possess precise predictive knowledge regarding outcomes, probabilities, or quality‐of‐life consequences. Clinical judgement therefore cannot function as a purely actuarial exercise. While medicine undeniably contains consequentialist structures, therapeutic ethics ultimately requires richer frameworks capable of incorporating uncertainty, patient individuality, proportionality, and practical wisdom.

### 3.8 Aristotle, Phronesis, and Clinical Judgement

Aristotelian virtue ethics may provide a more clinically realistic framework than strict utilitarianism. Aristotle emphasised phronesis, or practical wisdom, as the ability to navigate complex situations through contextual judgement rather than rigid rule application [28].

This framework aligns closely with actual medical practice. Physicians constantly navigate competing risks, incomplete information, and patient‐specific variables. The ethical task is not absolute avoidance of harm, but proportionate and context‐sensitive decision‐making.

Aristotle's doctrine of the mean is also relevant. Ethical medical practice exists between the extremes of therapeutic nihilism and reckless interventionism. Excessive avoidance of harm can itself become harmful if it results in undertreatment, while excessive intervention risks disproportionate injury.

Within this framework, the physician is not a technician applying protocols but a practitioner exercising disciplined judgement in contexts of vulnerability and moral complexity.

### 3.9 Kantian Ethics and Therapeutic Injury

The Kantian problem is more difficult than the presence of harm. Kantian ethics does not prohibit all injury; rather, it prohibits treating persons merely as means to an end. This distinction becomes especially complex in medicine, where therapeutic interventions frequently invade bodily integrity and intentionally destroy tissue [29].

As argued above, constitutive therapeutic injury cannot be redescribed as a foreseen side effect: the injury is the means by which the benefit is produced. The Kantian question is therefore posed in its sharpest form. If the destruction of tissue is intended as a means, is the patient thereby used as one?

Moral legitimacy therefore depends upon whether the patient remains an autonomous rational participant in the decision‐making process. Informed consent becomes central because it transforms therapeutic injury from unilateral bodily violation into voluntarily accepted intervention pursued in accordance with the patient's own rational goals and values.

Within this framework, foreseeable therapeutic injury may remain compatible with Kantian ethics provided the patient is never reduced to a passive object of technical manipulation or sacrificed solely for external ends such as institutional efficiency, physician ambition, or aggregate social utility.

### 3.10 Rossian Ethics and Practical Adjudication

Rossian ethics may ultimately describe clinical practice more accurately than either strict utilitarianism or rigid deontology. Ross recognized that moral life consists not of absolute rules but of competing prima facie duties requiring contextual adjudication [30].

In medicine, physicians simultaneously confront obligations of non‐maleficence, beneficence, autonomy, fidelity, truthfulness, and justice. These duties frequently conflict. Preventing neurological deterioration may require accepting procedural deficit risk. Respecting autonomy may require honoring decisions physicians believe medically unwise.

The adjudicative factors outlined in the proportionality analysis—severity of harm, probability of benefit, reversibility, disease trajectory, patient goals, alternatives, and prognosis—become operationalized through clinical judgement itself. Here Ross converges with Aristotle's concept of phronesis: Ross explains why duties conflict, while Aristotle explains how experienced practitioners navigate those conflicts through practical wisdom [31].

Ethical medicine therefore emerges neither from rigid rule application nor pure outcome maximisation, but from disciplined judgement exercised within contexts of uncertainty, vulnerability, and competing obligations.

### 3.11 Objections and Replies

Four objections deserve a direct reply. The first is that comparative harm management is simply consequentialism in clinical dress, and inherits its defects—above all the permissibility, in principle, of sacrificing one patient for greater aggregate good. The reply is that the account is agent‐ and patient‐relative in a way pure consequentialism is not: the harms and benefits weighed are, in the central case, borne by the same person, and the weighing is constrained by that person's consent and values. The view is therefore closer to the threshold‐bounded deontology described above than to welfare maximization across persons.

The second objection runs the other way: if harm is so readily justified, non‐maleficence has been emptied of content. This conflates the principle's operational and regulative roles. Non‐maleficence no longer functions as a literal prohibition, but it continues to discipline practice as a default and a burden of justification. The physician who would impose injury must show that a proportionate good is sought, that less injurious routes are unavailable, and that the patient consents. The principle survives precisely as the standing demand for that justification.

The third objection is that the account licenses therapeutic aggression. But the proportionality conditions are restrictive, not permissive: they forbid injury that is disproportionate, avoidable, non‐consensual, or pursued for ends external to the patient's good—institutional throughput, professional ambition, or aggregate utility. Properly stated, comparative harm management is at least as constraining as the literal principle it replaces, and considerably more honest about what medicine already does.

The fourth objection concerns what the account is for. If comparative harm management names something clinicians already do, it may seem to describe practice without altering it. The reply is that misdescription has costs, and that they fall on identifiable people. A physician who understands non‐maleficence as a prohibition, and who nonetheless burns, embolizes, resects or poisons as a matter of daily routine, is placed in the position of transgressing a rule she has been taught is foundational. The discrepancy does not remain theoretical. It is reported as unease at having caused injury the patient did not have before the encounter, as difficulty in distinguishing a complication from a completed treatment, and as reluctance to describe one's own work in the terms that would make it intelligible to a colleague or to a family.

Naming the structure does not dissolve that difficulty, but it relocates it. On the account offered here, the clinician who ablates a tumour is not violating non‐maleficence and justifying the violation; she is doing what the principle, properly stated, requires—choosing the configuration of harms that leaves the patient better placed than the alternatives, including the alternative of leaving the disease untreated. Distress that arises from believing one has broken a rule is answered differently from distress that arises from a hard choice made well. The first is dissolved by an accurate description of the act; the second is not, and should not be. An account that separates them allows institutions and colleagues to respond to each appropriately, rather than treating every form of discomfort at therapeutic injury as an individual failure of resilience.

## 4 Conclusion

Modern medicine routinely accepts foreseeable and sometimes near‐certain harm as an intrinsic component of therapeutic action. Controlled tissue injury, systemic toxicity, ischemia, bleeding risk, and neurological deficits are frequently anticipated aspects of therapy itself.

This reality fundamentally challenges literal interpretations of ‘primum non nocere.’ Contemporary medicine does not function through the elimination of harm, but through the ethical justification of proportional and comparative harms.

The analysis further suggests that medicine is structurally defined by morally asymmetric bodily relationships in which clinicians are granted socially sanctioned authority to invade bodily integrity, impose suffering, and produce injury in pursuit of therapeutic goals. These asymmetries may become even more complex as algorithmic systems increasingly participate in clinical decision‐making, potentially redistributing responsibility and complicating traditional understandings of clinical judgement, informed consent, and professional accountability.

The enduring value of ‘primum non nocere’ may therefore lie less in its literal operational accuracy than in its symbolic and cautionary function. The principle remains ethically valuable as a reminder against unnecessary injury and therapeutic excess, but it no longer accurately describes the operational morality of contemporary medicine. The physician functions not as a guarantor of innocence from harm, but as a steward of proportionate therapeutic injury operating within conditions of uncertainty, vulnerability, and competing moral obligations.

## Funding

The author has nothing to report.

## Ethics Statement

This article is a work of conceptual and normative analysis. It did not involve human participants, human tissue, personal data, or animals, and therefore did not require review by an institutional review board or research committee. No patient cases are drawn from the published literature.

## Conflicts of Interest

The author declares no conflict of interest.

## Generative AI & Authorship

Artificial intelligence (Claud Anthropic) has been used for language editing only. All content was generated by Boris Nikolic, MD, MBA.

## Data Availability

Data sharing is not applicable to this article, as no new data were created or analysed.
